# A Comparison Study of Precipitation in the Poyang and the Dongting Lake Basins from 1960–2015

**DOI:** 10.1038/s41598-020-60243-8

**Published:** 2020-02-25

**Authors:** Ruifang Guo, Yaqiao Zhu, Yuanbo Liu

**Affiliations:** 1 State Key Laboratory of Lake Science and Environment, Nanjing Institute of Geography and Limnology, Chinese Academy of Sciences, Nanjing, 210008 China; 2 Inner Mongolia Normal University, Hohhot, 010022 China; 3 Hubei Normal University, Huangshi, 435002 China

**Keywords:** Environmental sciences, Hydrology, Limnology

## Abstract

The Dongting Lake Basin and the Poyang Lake Basin, both located in the middle reaches of the Yangtze River, provide 30% of the total water volume for the Yangtze River. Under global climate change, precipitation patterns have undergone varying degrees of changes in different regions. Analysing temporal and spatial rainfall variations is important for understanding the variations in capacity of the two lake basins and the water intake variations by the Yangtze River. This study analyses the temporal and spatial variations of the two basins based on 33 rain-gauge data series from 1960–2015, using statistical methods, GIS spatial analysis and the M-K trend test. Our results showed that the annual precipitation generally increased in the Poyang Lake Basin and we found no obvious changes in the Dongting Lake Basin from 1960 to 2015. Seasonal precipitation levels at interannual scales were roughly consistent, but exhibited variability larger by an order of magnitude in the Poyang Lake Basin than in the Dongting Lake Basin. In general, an increasing trend dominated in spring and autumn while a decreasing trend was observed in summer and winter. The increasing trend was significant from the 1990s in the Poyang Lake Basin and from the late 1990s in the Dongting Lake Basin. It was found that annual precipitation with relatively larger anomalies appeared in ENSO (El Niño and Southern Oscillation) years in most cases, such as in 1963, 1997/1998 and 2002, while a few anomalies appeared in the previous or next year around an ENSO year, such as 1971 and 1978. All monthly precipitation periods with relatively larger or smaller anomalies coincided with ENSO events. In addition, El Niño and SOI (Southern Oscillation) events had significant relationships with negative monthly precipitation anomalies. El Niño and the SOI exerted more significant impacts on the Poyang Lake Basin than on the Dongting Lake Basin, which explains the conclusions regarding seasonal precipitation trends as mentioned above.

## Introduction

Due to global climate change, precipitation patterns have displayed varying degrees of changes in different regions^1,2^. These changes have effects on the temporal and spatial distribution of regional climates and on the environment, including water resources and human economic activities^3–5^. Changes in precipitation are one of the most important factors for assessing the potential for regional flooding^6–8^, drought risks^9–11^ and sustainable development^12^. Therefore, it is particularly important to compare temporal and spatial precipitation patterns in different regions.

Precipitation is the main source of water resources, and its changes result in distinct changes in runoff; the water volumes flowing into lakes then affect the water exchanges between lakes and rivers. The Yangtze River, China, is the third largest basin, and its precipitation changes have complex influences on water balance^13–15^. Dongting Lake and Poyang Lake are located in the middle and lower reaches of the Yangtze River. They play an effective role as regulators of the water capacity of the Yangtze River^16,17^. Their runoffs are 1647 × 10^8^ m^3^ and 1453 × 10^8^ m^3^, respectively, comprising 17.3% and 15.3% of the total runoff of the Yangtze River, respectively. It is important to study the trends and change regimes of precipitation in the two basins and their relationships with teleconnection modes to understand water changes and the water supply for the Yangtze River.

Many studies have reported on the spatial and temporal trends in the Dongting Lake Basin and the Poyang Lake Basin. For the Dongting Lake Basin, Wang *et al*.^18^ studied the spatial and temporal characteristics of precipitation from 1961–2003 using 22 rain gauge data measurements. Li *et al*.^19^ studied the spatial and temporal characteristics of precipitation from 1960–2008 with 27 rain gauge data measurements. Wang *et al*.^20^ studied multi-scale characteristics and trend predictions for precipitation from1958–2009 using 11 rain gauge data measurements. Xu *et al*.^21^ studied the spatial and temporal characteristics of precipitation from1960–2011 using 26 rain gauge data measurements. For the Poyang Lake Basin, Guo *et al*.^22^ studied the trends and abrupt changes in precipitation from1961–2003 using 14 rain gauge data measurements. Zhan *et al*.^23^ studied the changes in precipitation trends from 1959–2008 using 14 rain gauge data measurements. Yuan *et al*.^24^ studied the trends in precipitation changes from 1960–2007 using 49 rain-gauge data measurements.

The two lakes studied are located in the middle and lower reaches of the Yangtze River, and are in a humid subtropical climate zone. Many researchers have noted that precipitation has shown an increasing trend in the Yangtze River Basin. The two lake basins are at the centre of the increased precipitation^21,25–27^. However, these results have poor comparability over different study periods, datasets and methods in the two lake basins. It is difficult to obtain consistency and difference measurements for the precipitation changes between the Dongting Lake Basin and the Poyang Lake Basin. Therefore, it is necessary to compare the temporal and spatial characteristics of the two lake basins using the same datasets and methods. Especially, due to global climate warming, precipitation produces varying degrees of change for different regions over different periods, and varies for varying lengths of time. Precipitation influences the utilization of regional water resources. It is necessary to understand the basic changes in the water supplied from the two lakes to the Yangtze River under the conditions of global climate change. This study focuses on the comparisons of precipitation changes between the Dongting Lake Basin and the Poyang Lake Basin. This study expects to provide decision support information that is useful for regional water resource management and utilization in the Yangtze River Basin.

This study analysed the trends and regime changes in annual and seasonal precipitation and their relationships with teleconnection modes, using statistical methods, the M-K trend test and GIS spatial analysis tools with 33 rain-gauge data series and the teleconnection modes from 1960–2015 in the Dongting Lake Basin and the Poyang Lake Basin. To analyse the trend patterns, we first present the results of the temporal and spatial trends for annual and seasonal precipitation. We then study the correlations between precipitation and the teleconnection modes.

Table 1 provides the statistical characteristics of annual and seasonal precipitation from 1960–2015. In the Poyang Lake Basin, the average annual precipitation was 1631 mm with a SD (standard deviation) of 286 mm and a CV (coefficient of variance) of 0.18. The maximum annual precipitation was 2216 mm in 2012, while the minimum annual precipitation was 1059 mm in 1963. The CV of the annual precipitation ranged from 0.22 to 0.24 in the 1970s and 2000s, while it was 0.12–0.14 in other decades. In the Dongting Lake Basin, the average annual precipitation was 1451 mm with a SD of 169 mm and a CV of 0.12. The maximum precipitation was 1987 mm in 2002, while the minimum precipitation was 1028 mm in 2011. The CVs of precipitation were relatively low for every decade, and the largest CV values (0.16) occurred from 2000–2010. Obviously, precipitation and its spatial variability were higher in the Poyang Lake Basin. Furthermore, the timing of the year with the maximum of precipitation differed largely between the two basins.

**Table 1:** Statistical characteristics of the annual and seasonal precipitation from 1960–2015 for the Poyang Lake Basin and Dongting Lake Basin.

	Poyang Lake Basin					Dongting Lake Basin				
	Mean/mm	SD/mm	CV	Max/mm	Min/mm	Mean/mm	SD/mm	CV	Max/mm	Min/mm
Annual	1631	286	0.18	2217	1059	1452	170	0.12	1984	1028
Spring	639	138	0.22	970	290	502	88	0.17	739	253
Summer	557	138	0.25	904	290	498	118	0.24	778	270
Autumn	451	93	0.42	451	72	246	79	0.32	422	95
Winter	214	77	0.36	411	67	178	48	0.27	271	69

**Figure 1:**
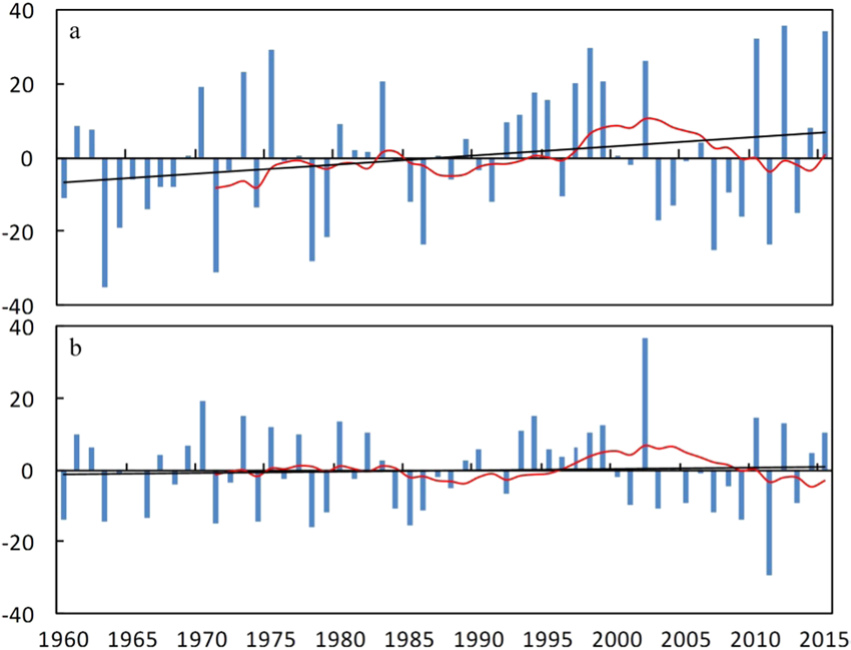
The time series of the area-weighted normalized annual precipitation anomalies relative to the 1960–2015 mean value (as percentages) for (**a**) the Poyang Lake Basin (**b**) the Dongting Lake Basin. The bars represent the normalized precipitation anomaly values, the red curves show five-year running means and the black lines are the linear regression lines.

Fig. 1 shows time series of the area-weighted normalized precipitation anomalies relative to the 1960–2015 mean value for the Poyang Lake Basin and the Dongting Lake Basin. The most striking feature is the strong variability at both interannual and interdecadal scales. In the Poyang Lake Basin, the precipitation anomalies increased at a rate of 3% decade^−1^. The normalized precipitation anomalies ranged from −35% (1963) to 36% (2012). The years 1975 (29%), 1998 (30%), 2010 (32%), 2012 (36%), and 2015 (34%) were very wet, while the years 1963 (−35%), 1971 (−31%), 1978 (−28%) and 2007 (−25%) were very dry. The 1960s and the 2000s were mostly dry, and the 1990s were mostly wet. The period from the 1970s to the 1980s and after 2010 was generally alternately wet and dry. In the Dongting Lake Basin, precipitation anomalies increased at a rate of 0.33% decade^−1^. The area-weighted normalized precipitation anomalies ranged from −29% (2011) to 37% (2002). The years 1970 (19%), 1973 (15%), 1994 (15%) and 2002 (37%) were very wet, while the years 1963 (−14%), 1978 (−16%), 1985 (−15) and 2011 (−29) were very dry. Precipitation maintained a recurring plus-minus anomaly pattern from the 1960s to the 1980s and after the 2010s. The period between the late 1980s and the early 2000s, and especially the 1990s, were mostly wet, and the early 2000s were mostly dry. Therefore, it was confirmed that the spatial variability in precipitation were higher in the Poyang Lake Basin and characteristics, such as the trend and variability in precipitation at interannual and interdecadal scales differed greatly between the two basins.

**Figure 2:**
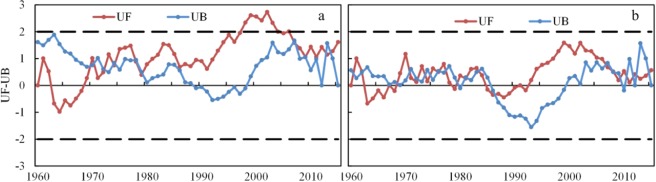
Trends and regime changes of the annual precipitation in (**a**) the Poyang Lake Basin (**b**) the Dongting Lake Basin from 1960–2015.

Figure 2 shows the trends and regime changes for the annual precipitation in the two basins. Generally, annual fluctuations in precipitation have been large for both basins, and precipitation showed no significant trends or abrupt changes. In the Poyang Lake Basin, the UF values became positive from the 1970s onwards, indicating a decreasing trend for precipitation until then, followed by an increase in precipitation. The UF was statistically significant at a confidence level of 0.95 from 1997 to 2006, indicating that the increasing trend was significant. Precipitation exhibited increasing and decreasing phases, suggesting that the basin was alternatively dry and wet in different periods. Specifically, there were three low precipitation periods from 1962–1968, 1984–1991 and 2003–2008, and there was one high precipitation period from 1992–2002. Precipitation showed no obvious trend for the periods from 1969–1983 and 2009–2015. In the Dongting Lake Basin, the UF values became positive from the 1970s, were negative from 1985 to 1992 and positive from 1993 onwards. It suggested that the Dongting Lake Basin showed the same trends as those of the Poyang Lake Basin. There was one high precipitation period from 1993–2010. The precipitation tended to alternatively increase and decrease during the other periods. Overall, annual precipitation exhibited increasing and decreasing phases in the two basins, but the trends did not reach significant levels except the periods from 1997 to 2006 in the Poyang Lake Basin. An increasing trend was determined from the 1970s onwards for the Poyang Lake Basin, while this trend was observed from 1993 onwards in the Dongting Lake Basin.

As seen in Table 1, the average seasonal precipitation levels were 639, 556, 451 and 213 mm in spring, summer, autumn and winter, respectively, and accounted for approximately 34%, 30%, 24% and 11%, respectively, of the annual total in the Poyang Lake Basin. The differences between the maximum and the minimum values were 680, 614, 381 and 343 mm in spring, summer, autumn and winter, respectively. The SD/CV values were 138 mm/0.22, 138 mm/0.25, 93.36 mm/0.42 and 77 mm/0.36 in the corresponding seasons. Obviously, autumn showed the largest CV, with winter showing the second highest CV and spring exhibiting the smallest CV, although the largest SD was observed in spring. It indicated that precipitation differences are greatest in autumn and the differences are smallest in spring. The average seasonal precipitation values were 502, 498, 246 and 178 mm, accounting for 35%, 35%, 17% and 13% of the annual precipitation in spring, summer, autumn and winter, respectively, in the Dongting Lake Basin. The differences between the maximum and the minimum values were 487, 508, 328 and 202 mm in spring, summer, autumn and winter, respectively. The SD/CV values were 138 mm/0.22, 138 mm/0.25, 93 mm/0.42 and 77 mm/0.36 in the corresponding seasons. The same relations were observed for the Poyang Lake Basin and the differences in precipitation were greatest in autumn, when precipitation was relatively low. Precipitation differences were lowest during spring, when precipitation was abundant. In general, the seasonal precipitation levels exhibited similar features in the two basins. Precipitation decreased from spring, summer, autumn to winter. The CVs were highest in autumn, followed by winter, spring, whereas the lowest CVs were found in spring.

**Figure 3:**
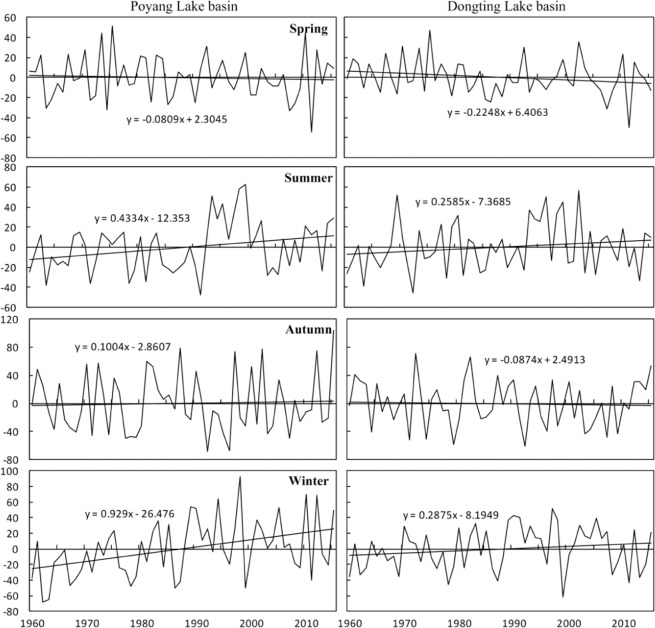
Time series of the area-weighted normalized seasonal precipitation anomalies relative to the 1960–2015 mean value (as percentages) for the Poyang Lake Basin and the Dongting Lake Basin. The curves represent the normalized seasonal precipitation anomaly values, and the black line is the linear regression line.

Figure 3 shows the time series of the area-weighted normalized seasonal precipitation anomalies relative to the 1960–2015 mean value for the two basins. Generally, the seasonal precipitation was not stable, and rising or falling trends existed for every season, especially in summer and winter. In the Poyang Lake Basin, the precipitation anomalies ranged from −55% (2011) to 52% (1975) in spring, −48% (1991) to 62% (1999) in summer, −68% (1992) to 104% (2015) in autumn and −68% (1962) to 92% (1998) in winter. Precipitation showed slight decreasing trend (−0.08 mm/year) in spring and an increasing trend (0.10 mm/year) in autumn. Overall, the precipitation anomalies were alternately positive and negative for these two seasons. Precipitation in summer and winter increased at rates of 0.43 mm/year and 0.93 mm/year, respectively. The period from the 1960s to the 1980s was mostly dry, and the 1990s were mostly wet in summer. The period after 2010 was generally alternately wet and dry. The period before the 1990s was mostly dry and after the 1990s was mostly wet in the winters. These trends indicated a redistribution of annual precipitation among different seasons. It was further confirmed that the annual precipitation increased. In the Dongting Lake Basin, the precipitation anomalies ranged from −50% (2011) to 47% (1975) in spring, −46% (1972) to 56% (2002) in summer, −61% (1992) to 72% (1972) in autumn and −62% (1999) to 52% (1997) in winter. Generally, precipitation showed similar traits to those determined for the Poyang Lake Basin, except for the winter season. The period from the 1990s to the late 2000s was mostly wet and the other periods were alternately wet and dry. These results indicated that the characteristics of seasonal precipitation at interannual scales were roughly consistent, but with a variability stronger by order of magnitude for the Poyang Lake Basin, than that observed in the Dongting Lake Basin. Furthermore, this result suggested that the change trend differed, especially in winter at interdecadal scales.

**Figure 4:**
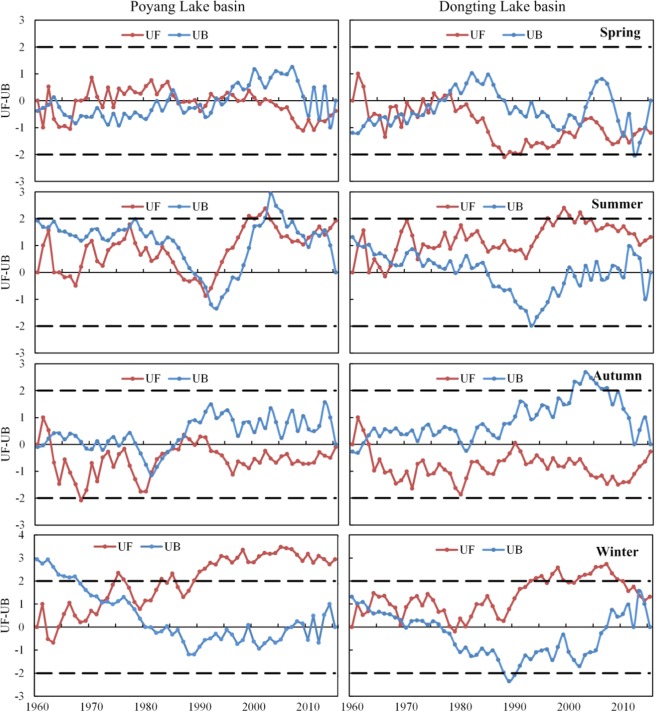
Trends and regime changes of seasonal precipitation in the Poyang Lake Basin and in the Dongting Lake Basin.

Figure 4 shows the trends and regime changes of seasonal precipitation in the two basins. In spring, the UF values became positive from the 1970s to the mid-1980s, and were negative after the 2000s in the Poyang Lake Basin. It indicated that precipitation decreased during the 1960s, had been on an upward trend from the 1970s to the mid-1980s, showed no change from the mid-1980s to the 1990s and then began to decrease after the 2000s. The UF values were negative, indicating that a decreasing trend was dominant in the Dongting Lake Basin. In summer, the UF values were positive except periods from 1985 to 1995 in the Poyang Lake Basin. It suggested that an increasing trend was dominant in the two basins except for a decreasing trend observed from 1985 to 1995. In contrast, the UF values were negative in autumn except periods from 1960 to 1965 in the two basins, and periods from 1985 to 1995 in the Poyang Lake Basin. It indicated that a decreasing trend was dominant except for an increasing trend from 1960–1965 in the two basins and during the period from 1985 to 1995 in the Poyang Lake Basin. In winter, the UF values were positive except after 2010 in the Dongting Lake Basin, indicating that an increasing trend was dominant except for a decreasing trend after 2010 in the Dongting Lake Basin. In particular, the UF was statistically significant at a confidence level of 0.95 from the 1990s in the Poyang Lake Basin and from the late 1990s in the Dongting Lake Basin. In a general, an increasing trend was dominant in spring and autumn while a decreasing trend was observed in summer and winter. The increasing trend reached a significant level from the 1990s in the Poyang Lake Basin and from the late 1990s in the Dongting Lake Basin

**Figure 5:**
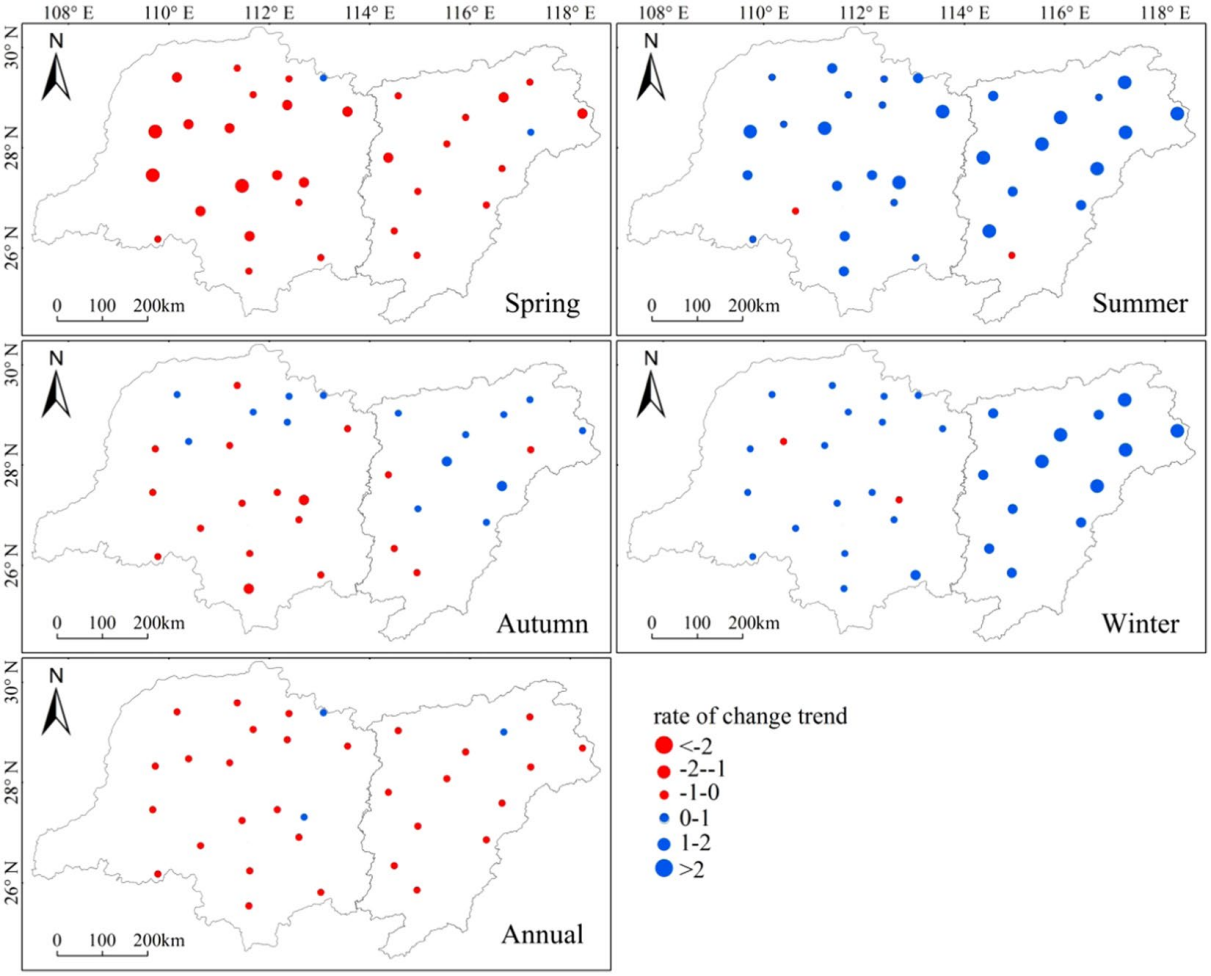
Spatial distribution of trends for winter, spring, summer, autumn and annual precipitation from 1951–2015. The figures were generated in ArcGIS (Geographic Information System) Software (version 10.1) (http://support.esrichina-bj.cn/).

Figure 5 displays the spatial distribution of the changing trends in annual and seasonal precipitation. The annual precipitation did not show any significant trend for the two lake basins as a whole; however, there were distinctive regional patterns. The annual total precipitation increased by 3–17 mm/decade in the Poyang Lake Basin and by 2–14 mm/decade in the Dongting Lake Basin.

For the Poyang Lake Basin, mostly positive trends were observed, except in spring. The change rates ranged from −3 to 51 mm/decade in summer, from −9 to 12 mm/decade in autumn and from 16 to 25 mm/decade in winter, whereas the rate fell from −18 to 4 mm/decade in spring. In summer, precipitation increased slightly in the water areas and in the southern basin. For winter precipitation, positive trends generally exhibited small changes in the southwestern part of the basin and in the water areas, whereas changes were large in the northeastern part of the basin. While precipitation decreased in spring, most change rates were lower than the rate of increase in summer and winter. In autumn, precipitation slightly increased in most regions except for the southwest basin. These results further suggested that the precipitation increased from a spatial standpoint. For the Dongting Lake Basin, mostly positive trends were observed in summer and winter, while mostly negative trends were seen in spring and autumn. The change rates ranged from −2 to 27 mm/decade in summer and from −2 to 10 mm/decade in winter, whereas the rates fell from −23 to 0.30 mm/decade in spring and from −20 to 10 mm/decade in autumn. For spring precipitation, the negative trends generally showed small changes in the southern, western and northeast basins, and exhibited large changes in other regions. In autumn, precipitation decreased in most regions, but increased slightly in the northwestern part of the basin. For summer precipitation, the trends were clearer for the mountainous regions. In winter, precipitation slightly increased in most regions. These results imply that a trend was not obvious in the Dongting Lake Basin.

In summary, negative trends appeared in most regions of the two basins with higher rates in the Poyang Lake Basin in spring. Precipitation increased more in the Poyang Lake Basin in summer and winter, and the rate of increase was greater for summer precipitation than for winter precipitation in the Dongting Lake Basin. In autumn, precipitation slightly decreased in most regions and the rate of decrease was rather equivalent between the two basins.

Tables 2 and 3 show the statistical correlations between climate teleconnections and monthly precipitation in the Poyang Lake Basin and in the Dongting Lake Basin. The SOI indices showed significant negative correlations in February and March in the Poyang Lake Basin, and in February and November in the Dongting Lake Basin. In general, positive relationships were dominant between Niño3.4 and the monthly precipitation values during October and March and negative relationships were dominant during April and September. The Niño3.4 indices showed significant positive correlations from October to March in the Poyang Lake Basin and from October to January in the Dongting Lake Basin. These results indicated that the SOI may bring less precipitation, especially in spring and winter, while Niño3.4 played an important role in increasing precipitation especially in winter. Furthermore, the Niño3.4 showed more prolonged influences on the Poyang Lake Basin. The NAO indices showed significant positive correlations in January and February and the PDO for the two basins exhibited significant positive correlations in July. As a whole, these climate teleconnection modes, especially for El Niño had impacts on precipitation in different seasons.

**Table 2:** The correlations between the area-averaged monthly precipitation and teleconnection modes in the Poyang Lake Basin.

	SOI		Niño3.4		NAO		PDO	
	*R*	*T* statistics	*R*	*T* statistics	*R*	*T* statistics	*R*	*T* statistics
Jan	−0.21	−1.43	0.25^*^	**2.11**	0.28^*^	**2.42**	0.05	0.36
Feb	−0.33^*^	−**2.09**	0.27^*^	**2.31**	0.31^**^	**2.75**	0.11	0.84
Mar	−0.48^**^	−**2.91**	0.31^*^	**2.77**	0.12	0.97	0.24^*^	**1.98**
Apr	−0.07	−0.52	−0.01	−0.05	−0.23	−1.50	0.00	−0.01
May	0.04	0.34	0.05	0.38	−0.18	−1.21	0.02	0.13
Jun	0.05	0.37	−0.05	−0.37	−0.02	−0.13	−0.01	−0.08
Jul	−0.09	−0.61	0.15	1.24	−0.18	−1.23	0.38^**^	**3.50**
Aug	−0.06	−0.42	−0.04	−0.29	−0.09	−0.62	−0.15	−1.00
Sep	0.04	0.33	−0.06	−0.41	−0.09	−0.61	−0.12	−0.80
Oct	−0.02	−0.16	0.26^*^	**2.25**	0.14	1.15	0.07	0.56
Nov	−0.21	−1.42	0.54^**^	**5.81**	0.00	−0.02	0.04	0.31
Dec	−0.14	−0.95	0.29^**^	**2.56**	0.00	−0.02	−0.15	−1.03

R is correlation coefficient. SOI is the Southern Oscillation. NAO is the North Atlantic Oscillation. PDO is the Pacific Decadal Oscillation.

**Table 3:** The correlations between the area-averaged monthly precipitation and teleconnection modes in the Dongting Lake Basin.

	SOI		Niño3.4		NAO		PDO	
	*R*	*T* statistics	*R*	*T* statistics	*R*	*T* statistics	*R*	*T* statistics
Jan	−0.19	−1.27	0.22^*^	**1.83**	0.23^*^	**1.93**	−0.03	−0.20
Feb	−0.19	−1.27	0.16	1.29	0.36^**^	**3.35**	0.11	0.89
Mar	−0.36^*^	−**2.28**	0.17	1.38	0.05	0.37	0.07	0.51
Apr	0.11	0.86	−0.12	−0.82	−0.23	−1.53	−0.18	−1.22
May	0.11	0.88	−0.09	−0.62	−0.10	−0.70	−0.10	−0.72
Jun	0.13	1.05	−0.03	−0.20	0.07	0.55	0.05	0.40
Jul	−0.17	−1.16	0.17	1.38	−0.10	−0.67	0.26^*^	**2.22**
Aug	0.08	0.60	−0.20	−1.33	−0.02	−0.11	−0.20	−1.35
Sep	0.02	0.16	−0.03	−0.23	−0.01	−0.07	−0.12	−0.86
Oct	−0.16	−1.10	0.27^*^	**2.34**	0.20	1.67	0.08	0.60
Nov	−0.32^*^	−**2.07**	0.54^**^	**5.77**	0.10	0.79	−0.02	−0.16
Dec	−0.18	−1.21	0.30^**^	**2.61**	−0.18	−1.19	−0.04	−0.29

The correlations between regional monthly precipitation and climate teleconnections for wet and dry months are shown in Table 4. Wet and dry months mean positive and negative monthly anomalies, respectively. Positive relationships were identified between the SOI and monthly precipitation for the wet months, whereas negative relationships were found for the dry months. In contrast, the Niño3.4 showed negative correlations for the wet months, whereas positive relationships were seen for the dry months. Significant correlations were found for the dry months in the Poyang Lake Basin between SOI/Niño3.4 and monthly precipitation. The relationships between the NAO and the PDO and monthly precipitation both did not reach significant levels. These results suggest that El Niño and the SOI had closer relationships with monthly precipitation with negative anomalies. In addition, El Niño and the SOI had significant impacts on the Poyang lake Basin compared to the Dongting Lake Basin.

**Table 4:** The correlations between regional monthly precipitation and teleconnection modes for wet and dry months in the two basins.

		SOI		Niño3.4	
		*R*	*T* statistics	*R*	*T* statistics
The Poyang Lake Basin	wet	0.05	0.35	−0.11	−0.74
	dry	−0.33^*^	**−2.10**	0.22^*^	**1.84**
The Dongting Lake Basin	wet	0.05	0.39	−0.17	−1.16
	dry	−0.17	−1.15	0.19	1.52

## Discussions and Conclusions

Precipitation and its spatial variability were higher in the Poyang Lake Basin at both the annual and seasonal scales. In addition, the years and months with maximum and minimum precipitation differed greatly. This implies that the precipitation in the two adjacent regions, despite having the same latitude and climate type, is probably characterized by strong variability. The differences between the two basins caused them to play different roles in river–lake water exchanges and in the discharges into the Yangtze River. In fact, the differences in precipitation characteristics between the two basins were due to their geographical locations. The southeastern monsoon played a key role in the precipitation changes in the two lake basins. The southeastern monsoon is strong as a result of land-sea pressure differences and thermal differences. However, the effect of the southeastern monsoon on the Dongting Lake Basin is weaker because it is farther away from the ocean. Because of the block of the Himalayas, the southwestern monsoon has little influence on the two lake basins, especially on the Poyang Lake Basin. Tables 5 and 6 show the statistical correlations of monthly and seasonal precipitation between the two basins. It is hardly surprising that the precipitation both at monthly and seasonal scales were highly correlated and reached a level of significance. The lowest *R*^2^ was in summer, because convective precipitation dominates and covers a smaller area, while frontal rains dominate in other seasons, especially in the spring. Therefore, the correlation is lowest in summer.

**Table 5:** The correlations of seasonal precipitation between the two basins.

	Spring	Summer	Autumn	Winter
*R*^2^	0.78	0.64	0.77	0.75

**Table 6:** The correlations of monthly precipitation between the two basins.

	Jan	Feb	Mar	Apr	May	Jun	Jul	Aug	Sep	Oct	Nov	Dec
*R*^2^	0.83	0.77	0.77	0.77	0.83	0.66	0.64	0.51	0.55	0.77	0.79	0.87

The annual precipitation anomalies showed a slight increasing trend in the Dongting Lake Basin, while they showed a relatively clear increasing trend (41 mm/decade, 3% decade^−1^) in the Poyang Lake Basin from 1960–2015, although they both did not reach a significant level. The staged trends from 1960–2015 were consistent with previous studies^21,28^. This trend resulted from both global climate change and the ENSO. Global precipitation generally increased especially in the 1990s and was the same in China and in the Yangtze River Basin^1,2,29^. ENSO is likely the largest source of inter-annual variability for the global climate^30^. Furthermore, the climate in China is greatly influenced by the East Asian monsoons^31–33^. It was found that there are notable interactions between ENSO and the East Asian winter monsoon^34,35^. It was also reported that the southeastern monsoon has become more intense due to global climate change^36^. From the results of the M-K trend tests, it is known that an increasing trend dominated from the 1970s in the Poyang Lake Basin while it dominated from 1993 in the Dongting Lake Basin and was alternatively dry and wet in other periods. These patterns further support the conclusions related to the increasing trends in annual precipitation and their differences.

The features of seasonal precipitation at interannual scales were roughly consistent, but exhibited a variability stronger by an order of magnitude in the Poyang Lake Basin than the Dongting Lake Basin. An increasing trend was dominant in spring and autumn, while a decreasing trend was present in summer and winter. The increasing trend reached a significant level starting from in the 1990s in the Poyang Lake Basin and from the latter part of 1990s in the Dongting Lake Basin. This consistent feature at interannual scales between the two basins is mainly due to the basins being in same climate zone. Negative trends appeared in most regions in the two basins, with higher rates in the Poyang Lake Basin in spring and autumn, while positive trends appeared in summer and winter. These results were also supported by examining the spatial distribution of trends. Various trends of different seasonal precipitation are presumably a response to ENSO, because ENSO had a stronger influence in autumn and winter. These findings for the seasonal changes were expected to provide evidence for changes in lake size, agriculture, flood and river-lake water exchanges and discharges in the Yangtze River.

There were some years and months with extreme positive and negative values. For annual precipitation, extreme positive values were observed in 1975, 1998, 2010, 2012, and 2015 in the Poyang Lake Basin, while they were observed in 1970, 1973, 1994 and 2002 in the Dongting Lake Basin. In contrast, extreme negative values appeared in 1963, 1971, 1978 and 2007 in the Poyang Lake Basin, while they appeared in 1963, 1978, 1985 and 2011 in the Dongting Lake Basin. For the monthly precipitation levels, extremely positive monthly values occurred in the spring of 1975, the summer of 1999, the autumn of 2015 and the winter of 1998 in the Poyang Lake Basin, while these extreme values were observed in the spring of 1975, the summer of 2002, the autumn of 1972 and the winter of 1997 in the Dongting Lake Basin. Extreme negative monthly values were seen in the spring of 2011, the summer of 1991, the autumn of 1992 and the winter of 1962 in the Poyang Lake Basin, while for the Dongting Lake Basin, these extreme negative values were observed in the spring of 2011, the summer of 1972, the autumn of 1992 and the winter of 1999. It is obvious that these extreme positive and negative values appeared mostly during ENSO events, such as the 1963, 1997/1998 and 2002 El Niño events^37^. A few cases appeared in the year before or after ENSO, such as in 1971 and 1978. Differing from the annual precipitation, all of the extreme monthly positive and negative values appeared during ENSO events. However, it is not evident whether El Niño had a significant relationship with high or low precipitation. It can be concluded that ENSO has a stronger influence in autumn and winter than in other seasons, which was reported by Wang and Li^30^. The positive annual values were related to positive monthly values, indicating that high monthly precipitation probably leads to high annual precipitation relative to the multi-year mean. However, the negative annual values were not significantly related to negative monthly values, indicating that low monthly precipitation levels do not lead to low annual precipitation relative to the multi-year mean. They imply that El Niño brings more rather than less precipitation. The differences between the two basins may be the result of varying degrees of the East Asian monsoons under ENSO conditions. The frequency and impact of extreme heat and heavy rainfall have increased in China^38^. Meanwhile, droughts, flood and typhoons have frequently occurred^39–41^. Our results may provide clues for the extreme changes that have occurred in the past 20 years. There is strong correspondence between ENSO and the extreme precipitation changes in the two basins.

Positive relationships are identified between the SOI and monthly precipitation for wet months, whereas there are negative relationships for the dry months. In contrast, Niño3.4 showed negative correlations for wet months, whereas there were positive relationships for the dry months. The Niño 3.4 Index represents average equatorial sea surface temperatures in the Pacific Ocean from about the International Date Line to the coast of South America. El Niño and the SOI had significant relationships with monthly precipitation exhibiting negative anomalies. In addition, El Niño and the SOI had more significant impacts on the Poyang lake Basin than on the Dongting Lake Basin, which explains the conclusions related to the seasonal precipitation trends as mentioned above.

### Study area and data

#### Study area

**Figure 6:**
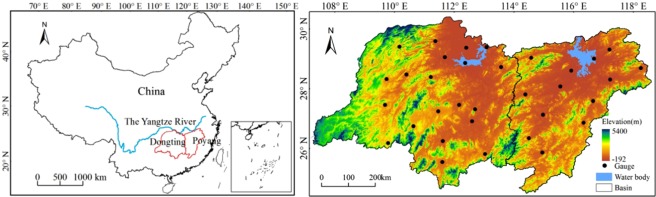
The locations of the Poyang Lake Basin and the Dongting Lake Basin (left), along with the surface elevations and the spatial distributions of 33 meteorological stations (right). The maps were generated in ArcGIS (Geographic Information System) Software (version 10.1) (http://support.esrichina-bj.cn/).

The Dongting Lake Basin lies between 24°38′ N to 30°26′ N and 107°16′ E to 114°17′ E. It has an area of 26.2 × 10^4^ km^2^ (Fig. 6). The Poyang Lake Basin (24°29′ N to 30°04′ N and 113°34′ E to 118°28′ E) is located to the east of the Dongting Lake Basin. Its area is 16.2 × 10^4^ km^2^. The two lake basins are located in the middle and lower reaches of the Yangtze River. They are located in a humid subtropical climate zone. Precipitation is unevenly distributed in space and time. Overall, 50% of the annual precipitation occurs from April to July, (the average annual precipitation from 1960–2010 was 1401/1636 mm in the Dongting Lake Basin/Poyang Lake Basin). Dongting Lake is the second largest freshwater lake in China. The surface elevation of the basin ranges from −26 to 2500 m above sea level. The southeastern basin is surrounded by mountains on three sides and forms a unique “horseshoe” pattern^42^. The Poyang Lake Basin is a part of the global terrestrial transects defined in the GCTE project of the IGBP^43^. The wetland is well known as the first batch of the Ramsar List of Wetlands of International Importance^44^. The surface elevation of the basin ranges from 5 to 2100 m above sea level. Most parts of the basin are dominated by hilly or mountainous topography. The area has great hydrological, biological, ecological, and economic significance.

#### Datasets

Rain gauge data are obtained from the National Meteorological Information Center of the China Meteorological Administration (CMA) (http://data.cma.cn/). The datasets consist of monthly precipitation records from 1960 through 2015 from 33 rain gauge stations, including 20 gauges in the Dongting Lake Basin and 13 gauges in the Poyang Lake Basin. A strict quality control process has been applied by the CMA to check and validate the extreme values^45^. Monthly data were accumulated to obtain the annual precipitation values. Then, the precipitation measurements for the basin are obtained by averaging the values using the Thiessen polygon method.

The regional climate is often driven by large-scale ocean–atmosphere interactions. Climate teleconnection modes are often used to describe the impact of larger climate oscillations on the regions climate^46–48^. They include the Niño3.4 index (sea surface temperature anomaly in the Niño3.4 region), the Southern Oscillation Index (SOI), the North Atlantic Oscillation (NAO) Index, and the Pacific Decadal Oscillation (PDO) Index. The Niño3.4 index is a measure of the sea surface temperature anomalies in the Niño3.4 region. The SOI is a standardized index based on the observed sea level pressure differences between Tahiti and Darwin, Australia. It is one measure of the large-scale fluctuations in air pressure that occur between the western and eastern tropical Pacific. El Niño and SOI are together called ENSO (El Niño and Southern Oscillation). The Pacific Decadal Oscillation (PDO) is often seen as a long-lived El Niño-like pattern of Pacific climate variability^49^. The North Atlantic Oscillation (NAO) index is based on the surface sea-level pressure differences between the Subtropical (Azores) High and the Subpolar Low. The NAO has positive and negative phases. The NAO exhibits considerable interseasonal and interannual variability^50^. These data are available from the National Oceanic and Atmospheric Administration–National Centers (https://www.ncdc.noaa.gov/teleconnections/)

## Methods

### M–K trend test

The M-K trend test was used in this study. The M–K trend test is a rank-based non-parametric test^51,52^. This test uses the number of inversions of pairs of objects that would be required to transform one rank order into another, and has wide applications^53,54^. The data were ranked according to time, and then, each data point was successively treated as a reference data point and was compared to all of the data points that followed in time^55^. For a monotonic trend in the time series {*x*_k_, *k* = 1, 2,…, *n*}, the M-K test is defined as

1 $${d}_{k}=\mathop{\sum }\limits_{i=1}^{k}{r}_{i}\,(2\le k\le n)$$

where

2 $${r}_{i}=\{\begin{array}{ll}+1 & {\rm{if}}\,{x}_{i} > {x}_{j}\\ 0 & {\rm{otherwise}}\end{array}(j=1,\,2,\,\mathrm{..}.,\,i)$$

The statistical index Z _k_ is defined as

3 $${Z}_{k}=\frac{{d}_{k}-E({d}_{k})}{\sqrt{{\rm{Var}}({d}_{k})}}$$

where E(*d*_k_)= *n*(*n* − 1)/4 and Var(*d*_k_) = *n*(*n* − 1)(2*n* + 5)/72. Positive Z values indicate a positive trend while negative values denote s a negative trend. Upon testing either increasing or decreasing monotonic trends, the null hypothesis was rejected for an absolute value of Z > Z_1−(*a*/2)_ obtained from standard normal cumulative distribution tables, where a is the desired significance level. We used a significance level of 0.05. The MK test requires the time series to be serially independent. The M-K test is applicable only when all the observations in a time series are serially independent.

The test consists of the graphical representation of two curves, UF and UB, computed in a similar manner. Generally, positive UF values represent an increasing trend, and negative ones mean a decreasing trend. The increasing or decreasing trend reaches a significant level when UF values are higher or lower than the critical line. Interaction of UF and UB means abrupt change point when it reaches a significant level.

### Statistical metrics

The arithmetic mean, SD and CV were used to study the temporal characteristics in the two lake basins from 1960–2015. SD quantifies the variation or dispersion of the values, and CV describes the standardized measure of the variation or dispersion. High SDs and CVs indicate larger dispersions. The coefficient of determination (*R*^2^) is a widely used index for effective assessment of consistency. It measures the degree of linear association between the estimates and measures. *R*^2^ ranges from 0 to 1.0. The larger *R*^2^ is, the better the performance of the regression.

## References

[1] Dore MHI (2005). Climate Change and Changes in Global Precipitation Patterns: What Do We Know?. Environ. Int..

[2] Wang Y, Cao MK, Tao B, Li KR (2006). The Characteristics of Spatio-temporal Patterns in Precipitation in China under the Background of Global Climate Change. Geogr. Res..

[3] Karl TR, Riebsame WE (1989). The Impact of Decadal Fluctuations in Mean Precipitation and Temperature on Runoff: A Sensitivity Study over the United States. Climatic Change.

[4] Li T, Gao Y (2015). Runoff and Sediment Yield Variations in Response to Precipitation Changes: A Case Study of Xichuan Watershed in the Loess Plateau, China. Water.

[5] Zhou Y, Shi C, Fan X, Shao W (2015). The Influence of Climate Change and Anthropogenic Activities on Annual Runoff of Huangfuchuan Basin in Northwest China. Theor. Appl. Climatol..

[6] Seiler RA, Hayes M, Bressan L (2002). Using the Standardized Precipitation Index for Flood Risk Monitoring. Int. J. Climatol..

[7] Chiang YM, Hsu KL, Chang FJ, Hong Y, Sorooshian S (2007). Merging Multiple Precipitation Sources for Flash Flood Forecasting. J. Hydrol..

[8] Leung LR, Yun Q (2009). Atmospheric Rivers Induced Heavy Precipitation and Flooding in the Western U.S. Simulated by the WRF Regional Climate Model. Geophys. Res. Lett..

[9] Piccarreta M, Capolongo D, Boenzi F (2004). Trend Analysis of Precipitation and Drought in Basilicata from 1923 to 2000 within a Southern Italy Context. Int. J. Climatol..

[10] Masson-Delmotte V (2005). Changes in European Precipitation Seasonality and in Drought Frequencies Revealed by a Four-century-long Tree-ring Isotopic Record from Brittany, Western France. Clim. Dynam..

[11] Gocic M, Trajkovic S (2013). Analysis of Precipitation and Drought Data in Serbia over the Period 1980–2010. J. Hydrol..

[12] Swann, A. L., Longo, M., Knox, R. G., Lee, E. & Moorcroft, P. R. The impact of Deforestation in the Amazon on Precipitation in Other Regions of Brazil: A Challenge for Sustainable Development?. Agu Fall Meeting (2013).

[13] Beardsley RC, Limeburner R, Yu H, Cannon GA (1985). Discharge of the Changjiang (Yangtze River) into the East China Sea. Cont. Shelf Res..

[14] Chen Z, Li J, Shen H, Wang Z (2001). Yangtze River of China: Historical Analysis of Discharge Variability and Sediment Flux. Geomorphology.

[15] Yang SL, Zhao QY, Belkin IM (2002). Temporal Variation in the Sediment Load of the Yangtze River and the Influences of Human Activities. J. Hydrol..

[16] Hu Q, Feng S, Guo H, Chen G, Jiang T (2007). Interactions of the Yangtze River Flow and Hydrologic Processes of the Poyang Lake, China. J. Hydrol..

[17] Ou C (2014). Evolution Characters of Water Exchange Abilities Between Dongting Lake and Yangtze River. J. Geogr. Sci..

[18] Wang GJ, Jiang T, Wang YJ, Yu ZY (2006). Characteristics of climate change in the Lake Dongting Basin (1961–2003). J. Lake Sci..

[19] Li JG, Huang SF, Li JR, Zang WB (2010). Spatial-temporal Characteristics of Precipitation in the Lake Dongting Basin from 1960 to 2008. J. China Inst. Water Hydropower Res..

[20] Wang SQ, Xue LQ, Wang LR, Liu YH, Duan X (2015). Multi-scale Periodic Features and Trend Prediction and Their Spatial Distribution Pattern of Precipitation in Dongting Lake Basin. J. Water Hydropower Rural China..

[21] Xu WH, Ge DX, Li N, Zhang SH, Peng H (2016). Characteristics of Precipitation Variation in the Dongting Lake Basin During 1960~2011. Wet land sci..

[22] Guo H, Jiang T, Wang G, Buda S, Wang YJ (2006). Observed Trends and Jumps of Climate Change over Lake Poyang Basin, China: 1961–2003. J. Lake Sci..

[23] Zhan M, Yin J, Zhang Y (2011). Analysis on characteristic of precipitation in Poyang Lake Basin from 1959 to 2008. Procedia. Environ. Sci..

[24] Yuan L, Yang G, Li H, Zhang ZX (2014). Rainfall Multiple Time Scale Variation Rule of Poyang Lake Basin in the Past 50 years. Resour. Environ. the Yangtze Basin.

[25] Buda S, Tong J, Shi YF, Becker S, Gemmer M (2004). Observed Precipitation Trends in the Yangtze River Catchment from 1951 to 2002. J. Geogr. Sci..

[26] Zhang YL, Gao JZ, Ding YG, Jiang T (2006). Analysis of Time-spatial Characteristics and Evolutional Trends of Summer Precipitation in the Yangtze River Catchment. J. Trop. Meteorol..

[27] Jiang T, Kundzewicz ZW, Su B (2008). Changes in Monthly Precipitation and Flood Hazard in the Yangtze River Basin, China. Int. J. Climatol..

[28] Zhang Q, Liu Y, Yang G, Zhang Z (2011). Precipitation and Hydrological Variations and Related Associations with Large-scale Circulation in the Poyang Lake Basin, China. Hydrol. Process..

[29] Zhang Q (2008). Spatial and Temporal Variability of Precipitation Maxima During 1960–2005 in the Yangtze River Basin and Possible Association with Large-scale Circulation. J. Hydrol..

[30] Wang, S. W. & Li, W. J. Climate of China. China Meteorological Press (2007).

[31] Liang XZ, Samel AN, Wang WC (1995). Observed and GCM Simulated Decadal Variability of Monsoon Rainfall in East China. Clim. Dynam..

[32] Glantz, M. H. Currents of Change-El Nino’s Impact on Climate and Society. Cambridge University Press (1996).

[33] Samel AN, Wang WC, Wang WC (1999). The Monsoon Rain Band over China and Relationships with the Rurasian Circulation. J. Climate.

[34] Lau, N. C. & Wang, B. Interactions between the Asian Monsoon and the El Niño/Southern Oscillation. In, The Asian Monsoon, Berlin, Praxos, 479–512 (2006).

[35] Wang, B. & Li, Tim. East Asian Monsoon and ENSO Interactions. ed. C. P. Chang, World Scientific Publishing Company Book Seris, **2**, 117–212. East Asian Monsoon (2004).

[36] Collins M (2010). The Impact of Global Warming on the Tropical Pacific Ocean and El Niño. Nat. Geosci..

[37] Ding R, Li J, Tseng Y, Sun C, Xie F (2017). Joint Impact of North and South Pacific Extratropical Atmospheric Variability on the Onset of ENSO Events. J. Geophys. Res. Atmos..

[38] Chen H, Sun J, Chen X, Zhou W (2012). CGCM Projections of Heavy Rainfall Events in China. Int. J. Climatol..

[39] Li J, Chen J (2004). Climatic Abrupt Change of China in 2010?. Digest of Sci. Technol..

[40] Jiao L (2009). Scientists Line Up Against Dam That Would Alter Protected Wetlands. Science.

[41] Kundzewicz, Z. W. *et al*. Flood Risk and Its Reduction in China. Adv. In Water Resour. **130**, 37–45, Published: AUG (2019).

[42] Dou, H. & Jiang, J. Dongting Lake. Hefei: University of Science & Technology China press (2000).

[43] Canadell JG, Steffen WL, White PS (2010). IGBP/GCTE Terrestrial Transects: Dynamics of Terrestrial Ecosystems under Environmental Change. J. Veg. Sci..

[44] Finlayson, M., Harris, J., McCartney, M., Young, L. & Chen, Z. Report on Ramsar Visit to Poyang Lake Ramsar Site, P.R. China. Report Prepared on Behalf of the Secretariat of the Ramsar Convention. 12–17 April 2010. Available online, http://www.ramsar.org/pdf/Poyang_lake_report_v8.pdf (2010).

[45] Ma YZ, Liu XN, Xu S (1998). The Description of Chinese Radiation Data and Their Quality Control Procedures. Meteorol.15 Sci..

[46] Trenberth KE (1998). Progress During TOGA in understanding and Modeling Global Teleconnections Associated with Tropical Sea Surface Temperatures. J. Geophys. Res. Oceans.

[47] Liu ZY, Alexander M (2007). Atmospheric bridge, oceanic tunnel, and global climatic teleconnections. Rev.Geophys..

[48] Zhou H, Liu Y (2017). Spatio-temporal Pattern of Meteorological Droughts and Its Possible Linkage with Climate Variability. Int. J. Climatol..

[49] Zhang Y, Wallace JM, Battisti DS (1997). ENSO-like Inter-decadal Variability: 1900–93. J. Clim..

[50] Hakkinen S (1999). Variability of the Simulated Meridional Heat Transport in the North Atlantic for the Period 1951–1993. J. Geophys. Res. Oceans.

[51] Kendall, M. G. Rank Correlation Methods (London: CharlesGriffin) (1975).

[52] Mann HB (1945). Non-parametric Tests against Trend. Econometrica..

[53] Wang W, Chen X, Shi P, Gelder AP (2008). Detecting Changes in Extreme Precipitation and Extreme Streamflow in the Dongjiang River Basin in Southern China. Hydrol. Earth Syst. Sci..

[54] Liu YB, Wu GP, Zhao XS (2013). Recent Declines in China’s Largest Freshwater Lake: Trend or Regime Shift?. Environ. Res. Lett..

[55] Douglas EM, Vogel RM, Kroll CN (2000). Trends in Floods and Low Flows in the United States: Impact of Spatial Correlation. Journal of Hydrology.

